# Activity of capecitabine for central nervous system metastases from breast cancer

**DOI:** 10.3332/ecancer.2023.1638

**Published:** 2023-11-23

**Authors:** Mariana Carvalho Gouveia, Cassio Murilo Hidalgo Filho, Raquel Andrade Moreno, Heitor Castelo Branco Rodrigues Alves, Aline Sgnolf Ayres, Laura Testa, Renata Colombo Bonadio

**Affiliations:** Instituto do Câncer do Estado de São Paulo, Universidade de São Paulo, Av Dr Arnaldo 251, Cerqueira Cesar, São Paulo 01246-000, Brazil; † This author contributed equally to this work; a https://orcid.org/0000-0001-8194-7594; b https://orcid.org/0000-0002-7046-0059; c https://orcid.org/0000-0002-7729-7418; d https://orcid.org/0000-0001-5042-4897; e https://orcid.org/0000-0001-6080-7429; f https://orcid.org/0000-0001-5818-922X

**Keywords:** capecitabine, breast cancer, CNS metastases, leptomeningeal carcinomatosis

## Abstract

**Purpose:**

Central nervous system (CNS) metastases are a significant burden in breast cancer (BC). Capecitabine is a frequent choice in this scenario, but data supporting its single-agent activity are scarce. We aimed to evaluate the intracranial efficacy of capecitabine in CNS metastases from BC.

**Methods:**

This retrospective cohort included patients with CNS metastases from BC treated with capecitabine at a single centre. Study endpoints were intracranial CNS objective response rate (CNS-ORR), intracranial CNS disease control rate (CNS-DCR), intracranial CNS progression-free survival (CNS-PFS) and overall survival (OS).

**Results:**

209 patients were included; 41.6% hormone receptor-positive HER2-negative (HR + HER2-), 33.9% human epidermal growth factor receptor 2 positive (HER2+), and 26.4% triple-negative breast cancer (TNBC). Radiotherapy was performed in 90.4% and CNS surgery in 27.5%. Among patients accessible for intracranial response, 3-month CNS-ORR and CNS-DCR were 41.6% and 81.2%. CNS-ORR was numerically higher among TNBC (61% versus 38% in HR + HER2-BC and 35% in HER2 + BC) (*p* = 0.194). When considering patients who were not evaluable at 3-month as non-responders, the 3-month CNS-ORR was 19.1% (18.4% in HR + HER2-, 18.3% in HER2+, and 21.6% in TNBC). Nevertheless, TNBC was associated with lower CNS-PFS (*p* < 0.001) and OS (*p* < 0.001). Median PFS was 8.3 months in HR + HER2-, 5.0 months in HER2+, and 3.0 months in TNBC. Median OS was 8.7, 9.1 and 4.5 months, respectively.

**Conclusion:**

Among patients with BC and CNS metastases accessible for intracranial response at 3 months, intracranial activity was observed with capecitabine. These patients have a poor prognosis regardless of the BC subtype, especially in scenarios where newer therapeutic options are unavailable.

## Introduction

Brain metastases are a significant burden in solid malignancies. Breast cancer (BC) is the second most common tumour associated with central nervous system (CNS) metastases. The risk for CNS involvement varies according to the immunohistochemical subtype. Approximately one-third to half of the patients with human epidermal growth factor receptor 2 positive (HER2+), one-third of those with triple-negative breast cancer (TNBC), and 15% with hormone receptor-positive, HER2-negative (HR + HER2-) metastatic BC subtypes will develop brain metastases during their respective course of illness [1].

The development of new systemic therapies for BC has resulted in a substantial increase in overall survival (OS) over the last two decades. Based on the known neurotropism of this disease, a higher absolute number of patients with disease progression to CNS is expected. This type of involvement is still related to poor prognosis and is a major life-threatening issue [2].

Local therapy such as whole-brain radiotherapy (WBRT), stereotactic radiosurgery (SRS) and neurosurgery have been the basis of brain metastases treatment [3, 4]. Due to their risk and toxicity, especially the neurocognitive decline related do radiation, and considering that additional local therapy becomes an unsuitable option for many patients, systemic treatment is an appealing alternative.

The CNS efficacy of new molecules, such as tucatinib and trastuzumab deruxtecan (T-Dxd), has been demonstrated for patients with HER2-amplified disease. Despite encouraging data demonstrating an intracranial response rate of up to 73.3% for trastuzumab deruxtecan in a phase II study, costs and access still limit the use of these drugs in clinical practice [5, 6]. Besides that, for HR + HER2- and TNBC, the efficacy of systemic therapy for CNS metastases seems limited. The oral chemotherapy capecitabine is a frequent choice for patients with CNS metastases in clinical practice due to reports of intracranial activity and penetration in CNS [7, 8]. However, clinical data supporting CNS activity are scarce. Only case reports and retrospective studies with a small number of patients have reported outcomes using capecitabine as a single agent. Therefore, it is necessary to investigate the performance of this drug in a larger sample of patients [9–15].

This study was specifically designed to evaluate the intracranial efficacy of capecitabine in brain metastases from BC.

## Methods

### Study design and participants

This was a retrospective cohort of patients with metastatic BC to CNS that received systemic treatment with capecitabine, treated between 2008 and 2021 at the Instituto do Câncer do Estado de São Paulo.

Patients who had received chemotherapy with capecitabine and had brain images (computed tomography or MRI) available for evaluation were initially screened. Patients were included if they had histologically confirmed BC with parenchymal CNS metastases confirmed by computed tomography, magnetic resonance, or leptomeningeal carcinomatosis (LC) established based on positive oncotic cytology in the cerebrospinal fluid and/or CNS imaging consistent findings. All patients received systemic treatment with capecitabine after diagnosis of CNS metastases. No other systemic treatment was administered alongside capecitabine for these patients.

Exclusion criteria were previous use of capecitabine in a palliative setting before diagnosis of CNS metastases or another invasive malignant tumour in the last 5 years, except non-melanoma skin neoplasm. Patients who met the eligibility criteria were selected, and records were reviewed for clinical and demographic characteristics, treatment received, and outcomes. Only patients with available imaging at 3 months were included in the analysis of intracranial response rate.

The following data were collected: age at diagnosis, histological subtype, initial Tumor Classification System (TNM) staging, histologic grade, clinical subtype (TNBC, HER2-positive, HR + HER2-) treatment performed for localised and metastatic disease, chemotherapy and endocrine therapy lines in the metastatic setting, clinical presentation at the diagnosis of CNS metastases, Eastern Cooperative Oncology Group performance status (ECOG-PS) at the diagnosis of CNS metastases, CNS radiation therapy data (WBRT, radiosurgery), neurosurgery data, date of the first cycle of capecitabine, best intracranial response 3 months after capecitabine initiation, date of capecitabine discontinuation, date of CNS progression and date of death. Data were collected using the RedCap program. CNS imaging were reviewed by three neuroradiologists to evaluate response according to Response Assessment in Neuro-Oncology (RANO) criteria [1]. The study was approved by the Institutional Review Board and Ethics Committee.

The study objective was to evaluate the effectiveness of capecitabine for CNS metastases from BC. The endpoints included CNS objective response rate (CNS-ORR) and CNS disease control rate (CNS-DCR) in 3 months, CNS progression-free survival (CNS-PFS) and OS.

### Statistical analysis

Descriptive analyses were used to present clinical, pathological and demographic data. Qualitative variables are presented as frequencies and percentages. Quantitative variables are presented using median and range. Categorical variables were compared using Fisher's exact test. Student *t*-test was used to analyse quantitative variables.

The Kaplan-Meier method was used to estimate survival curves, and the log-rank test was used to evaluate the difference between the curves. Univariate and multivariable Cox proportional hazard models were used to assess OS-associated factors. Factors with a *p*-value lower than 0.1 in the univariate analysis were selected for inclusion in the multivariable analysis.

For statistical significance, a *p*-value less than 0.05 was considered significant. The statistical analyses were performed using Stata Software, version 15.1 (StataCorp, Texas, USA, 2017).

## Results

### Patients’ characteristics

Among 1,319 patients selected for electronic chart evaluation, 209 met the study eligibility criteria. Baseline characteristics are summarised in Table 1. In the cohort, 91.8.% of the patients had ductal carcinoma, 37.3% had de novo metastatic disease, 41.6% had HR + HER2-, 33.9% HER2-positive and 24.4% TNBC. Most of them had ECOG 0-1 (56.9%) and had received <2 chemotherapy lines (78.4%) before capecitabine. Only 7.1% of the patients were asymptomatic when the CNS disease was diagnosed. The two main symptoms were headache in 34.4% and focal neurological deficit in 32%. Regarding the type of CNS disease, 74.6% had parenchymal metastases without LC; 37.8% had ≥5 brain lesions. Radiotherapy was performed in 90.4% of the cases, and CNS surgery in 27.7%. Among patients who underwent radiotherapy prior to capecitabine, the median time from radiotherapy to capecitabine initiation was 3.7 months (range 0.03–59.4). Thirty-seven patients (17.7%) started capecitabine within 1 month of radiotherapy.

### Efficacy analysis

#### CNS radiological response

Ninety-six patients were evaluable for CNS radiologic response at 3 months according to RANO criteria. Overall, 3-month CNS-ORR and CNS-DCR were 41.6% and 81.2%, respectively. CNS-ORR was numerically higher among TNBC (61% versus 38% in HR + HER2- and 35% in HER2+), although the difference was not statistically significant (*p* = 0.194). On the other hand, the 3-month CNS-DCR was numerically lower among TNBC. The 3-month CNS-ORR and CNS-DCR according to BC subtype are detailed in Table 2.

Some patients (*n* = 29) were not evaluable by RANO criteria due to lack of access to baseline or control imaging (imaging was performed, but was not available because of a radiology system error). The remaining patients (*n* = 84) did not undergo 3-month imaging probably due to clinical worsening or death before this time point. For instance, 45 patients (21.5% of the overall cohort) died within 3 months of capecitabine initiation. Considering the high proportion of patients without radiologic control due to clinical worsening or death, the proportions of CNS-ORR and CNS-DCR might be overestimated. In a worst-case scenario sensitivity analysis, when we consider all patients who were not evaluable at 3-month as non-responders, the 3-month intracranial response rate decreases to 19.1% (18.4% in HR + HER2-, 18.3% in HER2+, and 21.6% in TNBC).

#### CNS progression-free and OS

**Figure 1.**
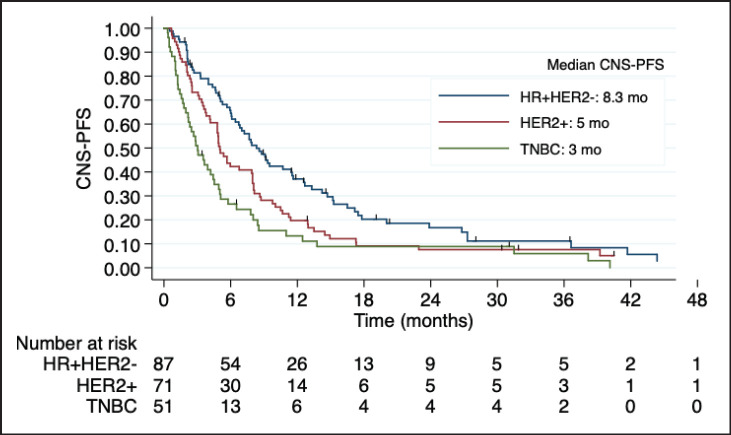
Kaplan-Meier estimates of CNS-PFS among BC subtypes, including table of median CNS-PFS according to subtype. CNS-PFS: Central nervous system progression free survival; HR + HER2-positive hormone receptor and negative human epidermal growth factor receptor 2; HER2+: positive human epidermal growth factor receptor 2; TNBC: triple negative breast cancer; mo: months; CI: Confidence interval; HR: Hazard ratio.

After a median follow up of 6 months, 185 patients presented CNS disease progression or death. The median CNS-PFS after capecitabine initiation was 5.6 months. Once again, outcomes were influenced by BC subtype. Patients with TNBC and HER2 + BC had a worse CNS-PFS compared with HR + HER2-BC (TNBC versus HR + HER2: HR 1.8; 95% CI 1.2 to 2.7; *p* < 0.001; HER2 + BC versus HR + HER2-BC: HR 1.45, 95% CI 1.0 to 2.0, *p* = 0.03). Median CNS-PFS was 8.3 months in HR + HER2-BC, 5.0 months in HER2 + BC, and 3.0 months in TNBC (Figure 1).

**Figure 2.**
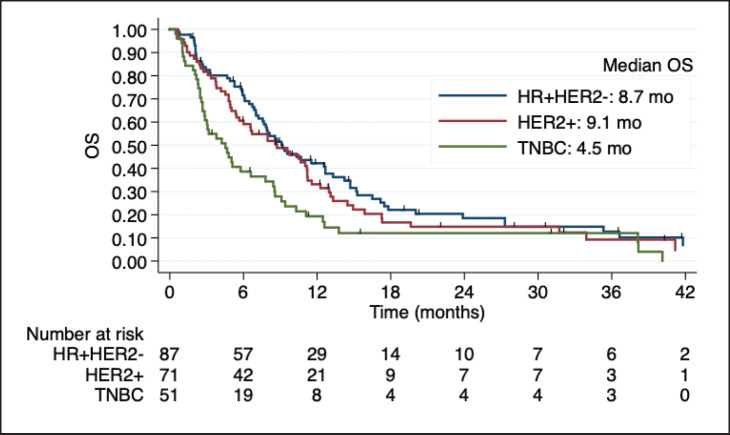
Kaplan-Meier estimates of OS among BC subtypes, including table of median OS according to subtype. OS: overall survival; HR + HER2-: positive hormone receptor and negative human epidermal growth factor receptor 2; HER2+: positive human epidermal growth factor receptor 2; TNBC: triple negative breast cancer; mo: months.

Median OS was 7.9 months in the overall cohort and was also lower in TNBC (4.5 versus 8.7 months in HR + HER2-BC and 9.1 months in HER2 + BC). The Kaplan- Meier curves of OS according to BC subtype, including detailed median OS, are presented in Figure 2. OS was also worse in patients with an ECOG-PS **≥**2 (*p* = 0.017) and was not influenced by the number or size of brain metastases, number of previous chemotherapy lines, and previous surgery or radiotherapy to CNS. Table 3 shows the factors associated with OS in the univariate Cox regression.

### Subgroup analysis of LC

**Figure 3.**
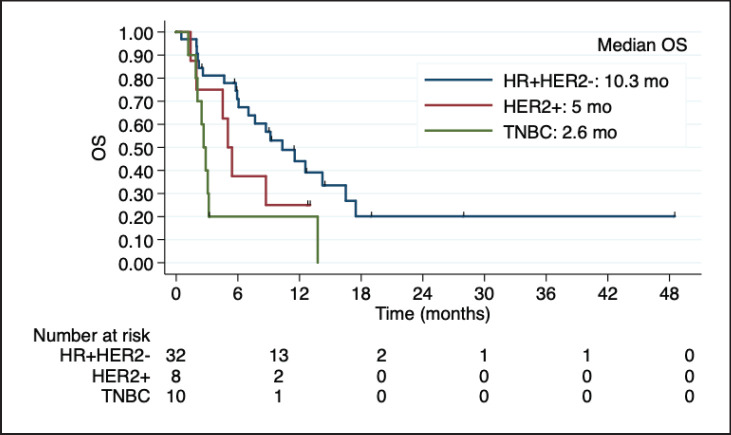
Kaplan-Meier estimates of OS among BC subtypes in patients with LC, including table of median OS according to subtype. OS: overall Survive; HR+HER2-: positive hormone receptor and negative human epidermal growth factor receptor 2; HER2+: positive human epidermal growth factor receptor 2; TNBC: triple negative breast cancer; mo: months.

Among 50 patients with LC, 29 (58%) had LC and parenchymal disease, and 21 (42%) had LC alone. Poor outcomes were observed in this subgroup, especially among HER2+ and TNBC (Figure 3). Indeed, in the univariate regression analysis, BC subtype was the only factor related to OS, with worse prognosis among TNBC (HR 3.94; 95% CI 1.69 to 9.19; *p* = 0.001).

## Discussion

To the best of our knowledge, this is the largest cohort evaluating capecitabine efficacy for CNS metastases from BC. Survival curves of the present study show that many patients present early disease progression and outcomes are overall poor. Nevertheless, approximately half of the patients were evaluable for radiological response at 3 months. Among these selected patients, promising data were observed for the three subtypes with satisfactory CNS-ORR (41.6%) and CNS-DCR (81.2%). Previous retrospective cohorts have suggested capecitabine activity in CNS metastases. An analysis of only seven patients with CNS metastatic BC treated with capecitabine reported encouraging results: three patients presented complete response, and three had stable disease in CNS [15]. Another retrospective study of 20 patients reported an overall intracranial response rate of 45% [16].

The development of new systemic therapies for BC has resulted in an unprecedented increase in the OS of these patients. However, the development and effectiveness of treatments for CNS have not advanced in the same way, with a few exceptions. However, the cost of some medications limits access in the public setting of low-middle income countries. For instance, tucatinib, trastuzumab deruxtecan (T-DXd), cyclin-dependent kinase inhibitors, and other novel treatments with CNS activity for metastatic BC are not available in the Brazilian public health system so far. Local therapy remains a standard treatment when feasible. In our study, 80.3% of the patients performed radiotherapy before capecitabine.

For HER2-positive patients some new therapeutic medications were developed with better evidence in CNS response. In HER2CLIMB, patients were randomly assigned to receive either tucatinib or placebo, in combination with trastuzumab and capecitabine. Among 291 patients with brain metastases not necessarily treated or controlled, the 1-year PFS was 24.9% in the tucatinib arm compared with 0% in the placebo-containing arm (hazard ratio 0.48, 95% confidence interval 0.34–0.69, *p* < 0.001). In an exploratory analysis of the subgroup of patients with brain metastases, the median intracranial confirmed objective response rate was 47% in the tucatinib arm versus 20% in the placebo arm (*p* = 0.03), with a median duration of intracranial response of 6.8 versus 3.0 months, and median OS of 18.1 versus 12.0 months, respectively [17]. Moreover, recent data from a single-centre phase II study showed promising intracranial response with trastuzumab deruxtecan, including patients with active brain metastases. TUXEDO-1 included 15 patients with HER2-positive metastatic BC and brain metastases that were either newly diagnosed or had developed upon disease progression after local therapy, previously treated with anti-HER2 directed therapy. The brain metastases were untreated in 40% of patients. Patients received treatment with T-DXd at 5.4 mg/kg every 3 weeks, yielding intracranial responses in 73.3% of the population and a median progression-free survival of 14 months [6]. Under the perspective of these new therapies, a response rate of 35% was observed in the HER2-positive group in our cohort. However, almost half of the patients were not evaluable at 3 months, some due to death or progressive disease. Also, the response was not durable, and these patients presented a poor prognosis illustrated by the mOS of only 9.1 months.

Regarding the HR + HER2- BC population, a phase II study showed the clinical activity of abemaciclib and pharmacokinetic results demonstrated relevant concentrations of abemaciclib and its active metabolites in brain metastases and cerebrospinal fluid. In the cohort of 58 heavily pretreated patients with brain metastases secondary to HR + HER2-metastatic BC, abemaciclib was administered twice daily as monotherapy or with endocrine therapy. In this cohort, three patients had confirmed intracranial partial responses, resulting in confirmed iORR of 5.2%. The intracranial clinical benefit rate was 25% (95% CI 13.1–35.2), the median intracranial PFS was 4.9 months, and the median OS was 12.5 months (95% CI, 9.3–16.4). In our analysis, a response rate of 38% was observed with capecitabine, but the median OS was only 8.7 months [18, 19].

TNBC patients had worst prognosis, similar to what happens without CNS involvement and to what is already known based on literature: the prognosis of CNS metastases from BC varies according to subtype. In previous data, for patients with TNBC with brain metastasis, mOS ranged from 2.9 to 4.9 months [20–22], in line with the mOS of 5.5 months here reported. The results reflect the still unmet need for better therapies for TNBC. The CNS efficacy of agents recently incorporated in TNBC treatment algorithm such as immune checkpoint inhibitors and sacituzumab govitecan remains to be determined (NCT04647916).

Our study has limitations due to its retrospective nature and the fact that almost half of the patients were not evaluable for radiologic response at 3 months mainly due to the poor prognosis of CNS metastases. Nevertheless, this is the largest study to evaluate capecitabine activity in BC in patients with CNS metastases. Our results reinforce that capecitabine seems to be active in CNS and is an option for systemic therapy in this scenario. Although the efficacy is influenced by the BC subtype, capecitabine can be considered an option regardless of the subtype. In fact, capecitabine can be a valuable option for HR + HER2- and HER2+ patients who do not have access to newer medications with CNS activity, such as tucatinib, T-Dxd and abemaciclib.

## Conclusion

The results suggest that patients with CNS metastases from BC still have a poor prognosis. TNBC subtype and ECOG-PS ≥2 were the main detrimental prognostic factors. TNBC was associated with worse long-term outcomes. Nevertheless, CNS-DCR was observed with capecitabine regardless of the BC subtype, suggesting some CNS activity of this drug, which might benefit selected patients in a limited sources scenario.

## Conflicts of interest

MCG, CMHF, RAM, HCBRA, ASF: No conflicts of interest. LT: Consulting or Advisory Role: Lilly, Novartis, MSD, AstraZeneca, Daiichi-Sankyo; Educational Support: Pfizer, Lilly, Zodiac, AstraZeneca; Speaker: Novartis, Roche, Pfizer, Zodiac, Lilly, MSD, AstraZeneca, Daichii-Sankyo; Institutional Research Funding: Novartis. RCB: Speaker fees and/or honoraria for consulting or advisory functions: Daiichi-Sankyo, Nestle; Financial support for educational programs and symposia: AstraZeneca, Daiichi-Sankyo; Institutional Research Funding: Novartis, AstraZeneca.

## Funding

This study was not supported by any grants or funding.

## Figures and Tables

**Table 1.** Patient demographic and disease characteristics (*N* = 209).

Median age (range)	49 (28–70)	
Histology – *n* (%)	Ductal	
Initial stage – *n* (%)	I	4 (19)
	II	28 (13.4)
	III	87 (41.6)
	IV	78 (37.3)
Histologic grade – *n* (%)	1	9 (4.3)
	2	78 (37.3)
	3	79 (37.8)
Subtype – *n* (%)	HER2+ HER2 + HR- HER2 + HR+	71 (33.7) 43 (20.5) 28 (13.3)
	HR + HER2-	87 (41.6)
	TNBC	51 (24.4)
ECOG – *n* (%)	0	39 (18.6)
	1	80 (38.2)
	2	38 (18.1)
	3 or more	22 (10.5)
Previous CT lines – *n* (%)	0	59 (28.2)
	1	101 (48.3)
	2	35 (16.7)
	3 or more	10 (4.8)
Previous hormone lines* – *n* (%)	0	91 (43.5)
	1	45 (21.5)
	2	34 (16.2)
	3 or more	35 (16.7)
Type of CNS metastases – *n* (%)	Parenchyma	156 (74.6)
	Leptomeningeal	21 (10)
	Both	29 (13.8)
Local treatment – n (%)	Surgery	58 (27.7)
	Radiotherapy	189 (90.4)
	WBRT only	127 (60.7)
	SRS only	27 (12.9)
	Both WBRT and SRS	35 (16.7)
	Previous to capecitabine After capecitabine	168 (80.3) 21 (10)

* Among hormone receptor-positive patients

N: Number; CNS: Central nervous system; ECOG: Eastern collaborative group; HER2+: HER2-positive; HR + HER2: hormone receptor-positive HER2-negative; TNBC: Triple negative breast cancer; HER2: Human epidermal growth factor receptor 2; CT, chemotherapy; WBRT: Whole brain radiotherapy; SRS: Stereotactic radiosurgery

**Table 2.** 3-month CNS-ORR and CNS-DCR (RANO criteria) (*n* = 96).

	HR + HER2- (N: 42)	HER2+ (*N*: 36)	TNBC (*N*:18)	*p*-value
CNS-ORR	38%	35%	61%	0.194
CNS-DCR	86%	80%	72%	0.428

CNS-ORR: Central nervous system objective response rate; CNS-DCR: Central nervous system disease control rate.; HR+: Positive hormone receptor; HER2-: Negative human epidermal growth factor receptor 2; HER2+: Positive human epidermal growth factor receptor 2; TNBC: Triple negative breast cancer

**Table 3.** Univariate and multivariable analysis of factors associated with OS among patients with CNS metastases from BC treated with capecitabine.

Variable	Univariate analysis			Multivariable analysis		
	HR	95% CI	*p* value	HR	95% CI	*p* value
Age (≥ 60 versus <60 years)	0.54	0.32–0.92	0.034	0.38	0.21–0.69	0.001
Subtype HR + HER2- HER2+ TNBC*	Ref. 1.16 1.87	0.82–1.65 **1.28–2.73**	0.384 **<0.001**	Ref. 1.22 2.02	0.83–1.80 1.33–3.06	0.305 0.001
ECOG-PS (2–4 versus 0–1)	1.53	1.07–2.17	0.017	1.81	1.24–2.66	0.002
BM size (>3 versus* ≤*3 cm)	0.84	0.59–1.20	0.352	-		
Number of CNS metastases (>4 versus* ≤*4)	1.22	0.89–1.68	0.202	-		
Radiotherapy (yes versus no)	0.91	0.48–1.74	0.792	-		
Radiotherapy timing (after capecitabine versus prior to capecitabine)	0.71	0.39–1.28	0.264	-		
Surgery (yes versus no)	0.74	0.53–1.04	0.088	0.60	0.40–0.90	0.014

HR: hazard ratio; CI: Confidence interval; HR+: Positive hormone receptor; HER2-: Negative human epidermal growth factor receptor 2; HER2+: Positive human epidermal growth factor receptor 2; TNBC: Triple negative breast cancer; BM: Brain metastases; CNS: Central nervous system

* TNBC subtype was statistically significant associated with worse overall survival
